# A retrospective cohort analysis of failure and potential causes after osteosynthesis of femoral fractures with VA-LCP Condylar plate 4.5/5.0, Depuy Synthes

**DOI:** 10.1016/j.jor.2024.04.004

**Published:** 2024-04-10

**Authors:** Nikolaj Hjort Schmidt, Lasse Birkelund, Jesper Ougaard Schønnemann

**Affiliations:** a Department of Orthopaedics, Hospital Sønderjylland, Kresten Philipsens vej 15, 6200, Aabenraa, Denmark; b Department of Orthopaedics, Odense Univesity Hospital, J. B. Winsløws vej 4, 5000, Odense C, Denmark

**Keywords:** Femoral fracture, Failure, Retrospective cohort analysis, VA-LCP Condylar plate 4.5/5.0, Trauma, Risk factors

## Abstract

**Background:**

Since 2014, the VA-LCP Condylar Plate 4.5/5.0, Depuy Synthes, has been the preferred implant for these injuries at our institution, however, speculations have been made whether it is more prone to failure compared to other implants. Thus, the aim of the study was to describe the cohort treated with the VA-LCP Condylar Plate 4.5/5.0, Depuy Synthes, at our department from 2014 to 2020, including the number of failures. Secondary, whether specific outcome measures were significantly overrepresented in the failure group.

**Methods:**

Patients were identified through the hospital database, and demographic data was extracted from patient files. X-rays were evaluated for injury type, osteosynthesis characteristics, and whether the construct failed during follow-up. Thanks to the national patient record database a minimum of patients was lost to follow-up.

**Results:**

After exclusion 159 patients (165 osteosyntheses, descriptive part) and 108 patients (112 osteosyntheses, subgroup analysis) were eligible for inclusion. The VA-LCP Condylar Plate 4.5/5.0, Depuy Synthes, was used for most AO-type fractures and inserted as both neutralization, buttress, and bridging plates. Overall failure was seen in 8 % of osteosyntheses. Significantly more failures were seen in patients with increased Body Mass Index (BMI) (24 vs. 32, p = 0,046) and those treated for a periprosthetic fractures (41 % vs. 89 %, p = 0,005). We did not see an association between failure and plate length, bridge span, screw density or the degree of medial support.

**Conclusion:**

The VA-LCP Condylar Plate 4.5/5.0, Depuy Synthes is a versatile plate with failure rates comparable to previously reported studies. This study confirmed that elevated BMI may be a risk factor for failure, while other previously reported risk factors were not associated with failure in this study.

## 1 Introduction

Femoral fractures need prioritized surgical attention. They occur with a bimodal distribution affecting both young and old patients often subjected to high- and low-energy trauma, respectively.^1^^,^^2^ The severity for these injuries is documented for the elderly, which suffer from a 10 % and 30 % mortality after 1 month and 1 year, respectively.^3^ Thus, for old fragile patients proper fixation that does not require reoperation seems obvious. However, in our department, approximately 20–30 patients are treated for AO/OTA 32 and 33-type injuries each year, which means that even when this type of surgery is only performed by a handful of surgeons, it is in our opinion never a routine procedure. Because of its versatile use, i.e., fixation of both naive and periprosthetic fractures, the VA-LCP Condylar Plate 4.5/5.0 (DePuy Synthes) has historically been the preferred implant at our institution since 2014 and until now for these types of injuries.

Due to a number of consecutive failures lately, concern was once again raised whether we, too, experience to high failure rates as described by Tank et al.^4^ Moreover, this reignited the long-standing discussion at our institution regarding construct design, plate flexibility, and whether short or long plates should be used. Gautier et al., and Stoffel et al., established simple versatile rules for the use of locking compression plates, i.e. rules we largely have adhered to.^5^^,^^6^ However, during the past years conflicting studies have once again raised the question whether these heterogenous injuries can be managed by “simple” rules since Ricci et al., found that shorter plate constructs retrospectively were associated to failure in contrast to McDonald et al., who found the opposite.^7^^,^^8^

Thus, to contribute to this rather low evidence area of research (i.e., studies are retrospective and with heterogenous injury types and patient population), we felt compelled to asses and describe our cohort of patients, including fixation constructs and the number of failures, treated with the VA-LCP Condylar Plate 4.5/5.0 (DePuy Synthes) from 2014 to 2020, which was the primary goal of this study. Secondary, whether some of these descriptive parameters were significantly overrepresented in the constructs that failed.

## 2 Methods

**Fig. 1:**
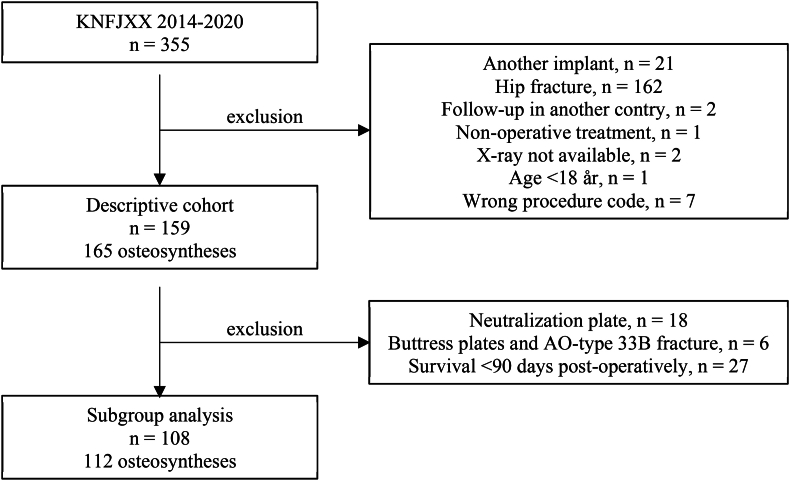
Flowchart for inclusion of patients.

Patients treated at our institution with a relevant procedure code (Appendix 1) from 2014 to 2020 were retrospectively identified through the regional patient record management system. Patients with hip fractures, aged <18, or who was not treated with the VA-LCP Condylar Plate 4.5/5.0 VA-LCP Condylar Plate 4.5/5.0 (DePuy Synthes) were excluded (Fig. 1). Data was acquired May 25, 2022 and patient records and x-rays were evaluated from August to December 2022. This study was approved by the Danish National Center for Ethics (Approved: 31-03-2022. File number: 2200031), the Executive Board at Hospital Sønderjylland (Approved: 22-04-2022), registered at The Region of Southern Denmark internal list of scientific studies (Approved: 05-04-2022. File number: 21/43666), and performed in compliance with relevant laws.

Demographic data (i.e., age, sex assigned at birth, Body Mass Index [BMI], Amercan Society of Anesthesiology score [ASA-score], diabetes, smoking), injury data (i.e., trauma-related energy, Gustillo-Anderson classification of open fractures), post-operative allowed weight-bearing, time to follow up or death, and post-operative signs of infection were acquired through review of patient records. X-rays were evaluated pre- and post-operatively for the injury type according to AO/OTA; periprosthetic fractures; medial comminution; the method of osteosynthesis (i.e., neutralization-, buttress- or bridge-plating); the ratios for plate-/fracture-length, bridge-/fracture-length, and bridge-/plate-length; the ratio of occupied screw holes both proximal and distal to the fracture as well for the entire plate; the use of supplemental cable, Locking Attachment Plates (DePuy Synthes) and/or medial plates; failure. To achieve near complete follow-up, The Danish National Record Register (where detailed patient records for every encounter the patient has with the healthcare system, i.e., all Danish hospitals, are kept) was reviwed to identify implant failure or signs of infection in patients who were followed up at other hospitals post-operatively.

All plate failures turned out to be bridging plates. In a subgroup analysis, matching controls were identified, i.e., patients operated with lag-screws and neutralization plates or buttress-plates (AO/OTA 33B-fractures), or patients who died <90 days after the primary procedure were excluded from the analysis (Fig. 1).

For continuous data, normal distribution was tested using the Shapiro-Wilk's test, and normally distributed data are reported as mean ± SD whereas non-parametric data are reported as median (IQR). For catagorial data, R x C tables are reported. Continuous data were tested using the unpaired *t*-test (normally distributed data) and the Wilcoxon rank sum test (non-parametric data). Categorical data with <40 observations or 0 observations in one of the spaces were tested using Fishers exact test. Other categorical data were tested using Pearson's Chi^2^-test. P < 0,05 is considered to be significant. Data were analyzed using Stata 13.0 (StataCorp LP, Texas, USA).

## 3 Results

For the descriptive part of the study, an initial search identified 355 patients of which 159 patients (165 osteosyntheses) were eligible for inclusion. After further exclusion, 108 patients (112 osteosyntheses, all bridging plates) were identified for the following subgroup analysis (Fig. 1).

Of the 165 osteosyntheses, 139, 20, and 6 were applied as bridging, neutralization, and buttress plates, respectively. Patient and osteosynthesis characteristics are shown in Table 1. Overall, plate osteosynthesis was performed for all AO type fractures (i.e., A, B, and C fractures), plate lengths were generally more than twice as long as the fractures, and it was only considered necessary to apply a medial plate in a small number of cases. For neutralization and bridging plates, the density of occupied screw holes was approximately 1/3, and there was an equal distribution of allowed post-operative weight-bearing in the two groups. However, it was mainly in the early years of the period, where the plate was still “new” in the department, that “no weigth-bearing” was instituted post-operatively, i.e., a precautionary approach may have been chosen until it empirically seemed okay to let the patients bear more weight. In line with expectations, patients treated with buttress plates were generally not allowed to bear weight post-operative, which in light of these intra articular fractures (all were AO-type B fractures) seems obvious.

**Table 1:** Patient and osteosynthesis characteristics.

	Bridging plates	Neutralization plates	Buttress plates
Number of osteosyntheses	139	20	6
Age, years, medial (IQR)	81 (17)	73 (24)	83 (11)
Sex			
Female	109 (78 %)	14 (70 %)	6 (100 %)
Male	30 (22 %)	6 (30 %)	0 (0 %)
BMI, median (IQR)	24 (8)	25 (4)	18(6)
ASA			
Not described, n	26 (19 %)	5 (25 %)	2 (33 %)
1, n	4 (3 %)	3 (15 %)	0 (0 %)
2, n	28 (20 %)	2 (10 %)	1 (17 %)
3, n	68 (49 %)	6 (30 %)	2 (33 %)
4, n	13 (9 %)	4 (20 %)	1 (17 %)
Diabetes			
No, n	116 (83 %)	18 (90 %)	5 (83 %)
Yes, n	23 (17 %)	2 (10 %)	1 (17 %)
Smoking			
No, n	87 (63 %)	12 (60 %)	3 (50 %)
Former, n	23 (17 %)	4 (20 %)	2 (33 %)
Current, n	29 (21 %)	4 (20 %)	1 (17 %)
Energy			
Prophylactic operation, n	1 (1 %)	0 (0 %)	0 (0 %)
Re-operation, n	1 (1 %)	1 (5 %)	0 (0 %)
Low, n	131 (94 %)	19 (95 %)	6 (100 %)
High, n	6 (4 %)	0 (0 %)	0 (100 %)
AO-type			
Prophylactic operation, n	1 (1 %)	0 (0 %)	0 (0 %)
32A1, n	41 (29 %)	8 (40 %)	0 (0 %)
32A2, n	9 (6 %)	1 (5 %)	0 (0 %)
32B1, n	0 (0 %)	1 (5 %)	0 (0 %)
32B2, n	18 (13 %)	0 (0 %)	0 (0 %)
32B3, n	1 (1 %)	0 (0 %)	0 (0 %)
32C3, n	1 (1 %)	0 (0 %)	0 (0 %)
33A2, n	31 (22 %)	6 (30 %)	0 (0 %)
33A3, n	22 (16 %)	0 (0 %)	0 (0 %)
33B1, n	0 (0 %)	3 (15 %)	5 (83 %)
33B2, n	0 (0 %)	0 (0 %)	1 (17 %)
33C1, n	9 (6 %)	1 (5 %)	0 (0 %)
33C2, n	5 (4 %)	0 (0 %)	0 (0 %)
33C3, n	1 (1 %)	0 (0 %)	0 (0 %)
Gustillo-Anderson classification			
Closed, n	136 (98 %)	20 (100 %)	6 (100 %)
1, n	1 (1 %)	0 (0 %)	0 (0 %)
2, n	2 (1 %)	0 (0 %)	0 (0 %)
3, n	0 (0 %)	0 (0 %)	0 (0 %)
Peri- and interprosthetic fracture			
No, n	75 (54 %)	11 (55 %)	5 (83 %)
Periprosthetic hip, n	39 (28 %)	6 (30 %)	1 (17 %)
Periprosthetic knee, n	16 (12 %)	3 (15 %)	0 (0 %)
Interprosthetic, n	9 (6 %)	0 (0 %)	0 (0 %)
Plate-/fracture length, median (IQR)	2,8 (1,8)	2,5 (1)	2,2 (1,2)
Bridge span/plate length, median (IQR)	0,5 (0,2)	–	–
Bridge span/fracture length, median (IQR)	1,3 (0,8)	–	–
Screw density in the plate			
Total, median (IQR)	36 % (19)	35 % (16)	83 % (50)
Proximal fragment, median (IQR)	75 % (40)	69 % (26)	92 % (33)
Distal fragment, median (IQR)	88 % (17)	83 % (24)	83 % (33)
Medial comminution			
Prophylactic operation, n	1 (1 %)	0 (0 %)	0 (0 %)
No, n	80 (58 %)	20 (100 %)	6 (100 %)
Yes, n	58 (42 %)	0 (0 %)	0 (0 %)
Use of cables			
No, n	53 (38 %)	17 (85 %)	6 (100 %)
Yes, n	86 (62 %)	3 (15 %)	0 (0 %)
Use of Locking Attachment Plate			
No, n	78 (56 %)	11 (55 %)	5 (83 %)
Yes, n	61 (44 %)	9 (45 %)	1 (17 %)
Use of medial plate			
No, n	137 (99 %)	20 (100 %)	6 (100 %)
Yes, n	2 (1 %)	0 (0 %)	0 (0 %)
Post-operative weight bearing			
None, n	50 (36 %)	6 (30 %)	4 (67 %)
Partiel, n	42 (30 %)	6 (30 %)	1 (17 %)
Full, n	47 (34 %)	8 (40 %)	1 (17 %)
Post-operative follow-up, days, median (IQR)	88 (201)	116 (287)	54 (67)
Death post-operative, days, median (IQR)	505 (1058)	22 (506)	1070 (1152)
Complications			
Infection, n	8 (6 %)	0 (0 %)	0 (0 %)
Failure, n	9 (6 %)	0 (0 %)	0 (0 %)
Time to failure, days (SD)	108 (77)	–	–

Failure was observed 9 times (all bridging plates) and documented with x-ray 108 ± 77 days after the primary osteosynthesis (characteristics are described in Appendix 2). The proportion of failures was 5,5 % (9/165) and 8 % (9/112) for the entire cohort and the subsequent subgroup analysis, respectively. In the subgroup analysis, three characteristics were significantly elevated in the failure group compared to controls, i.e., higher BMI among patients with failed implants, a larger proportion of initially treated periprostetic fractures, and elevated numbers of post-operative infections (Table 2). Neither plate- or bridge-span nor screw density were found to be independent risk factors for later failure. Patients were statistically allowed to bear equal amounts of weight in the two groups.

**Table 2:** Higher BMI among patients with failed implants, a larger proportion of initially treated periprostetic fractures, and elevated numbers of post-operative infections.

	**Controls**	**Failure**	
Number of osteosyntheses	103	9	
Age, years, medial (IQR)	79 (20)	78 (15)	p = 0,95 **‡**
Sex			
Female	85 (83 %)	9 (100 %)	p = 0,35 **†**
Male	18 (17 %)	0 (0 %)	
BMI, median (IQR)	24 (7)	32 (13)	**p = 0,046 ‡**
ASA			
Not described, n	20 (19 %)	3 (33 %)	p = 0,14 **⁎**
1–2, n	25 (24 %)	4 (44 %)	
3–4, n	58 (56 %)	2 (22 %)	
Diabetes			
Nej, n	88 (85 %)	6 (67 %)	p = 0,14 **⁎**
Ja, n	15 (15 %)	3 (33 %)	
Smoking			
No, n	61 (59 %)	7 (78 %)	p = 0,27 **⁎**
Former/current, n	42 (41 %)	2 (22 %)	
Energy			
Prophylactic operation, n	1 (1 %)	0 (0 %)	p = 0,36 **⁎**
Re-operation, n	0 (0 %)	1 (11 %)	
Low, n	97 (94 %)	7 (78 %)	
High, n	5 (5 %)	1 (11 %)	
AO-type			
Prophylactic operation, n	1 (1 %)	0 (0 %)	
32A1, n	32 (31 %)	1 (11 %)	p = 0,31 **†**
32A2, n	6 (6 %)	3 (33 %)	
32B2, n	16 (16 %)	0 (0 %)	
32B3, n	1 (1 %)	0 (0 %)	
33A2, n	18 (17 %)	3 (22 %)	p = 0,31 **†**
33A3, n	16 (16 %)	3 (33 %)	
33C1, n	8 (8 %)	0 (5 %)	
33C2, n	4 (4 %)	0 (0 %)	
33C3, n	1 (1 %)	0 (0 %)	
Gustillo-Anderson classification			
Closed, n	101 (98 %)	9 (100 %)	p = 0,67 **⁎**
1, n	0 (0 %)	0 (0 %)	
2, n	2 (2 %)	0 (0 %)	
3, n	0 (0 %)	0 (0 %)	
Peri- and interprosthetic fracture			
No, n	61 (59 %)	1 (11 %)	**p = 0,005 ⁎**
Periprosthetic hip, n	28 (27 %)	5 (56 %)	
Periprosthetic knee, n	7 (7 %)	3 (33 %)	
Interprosthetic, n	7 (7 %)	0 (0 %)	
Plate-/fracture length, median (IQR)	2,6 (1,9)	2,6 (0,9)	p = 0,66 **‡**
Bridge span/plate length, median (IQR)	0,5 (0,2)	0,4 (0,1)	p = 0,44 **‡**
Bridge span/fracture length, median (IQR)	1,3 (0,8)	1,2 (1,1)	p = 0,78 **‡**
Screw density in the plate			
Total, median (IQR)	36 % (22)	38 % (21)	p = 0,57 **‡**
Proximal fragment, median (IQR)	75 % (40)	71 % (19)	p = 0,58 **‡**
Distal fragment, median (IQR)	100 % (17)	83 % (29)	p = 0,18 **‡**
Medial comminution			
Prophylactic operation, n	1 (1 %)	0 (0 %)	p = 0,74 **⁎**
No, n	74 (71 %)	6 (67 %)	
Yes, n	29 (28 %)	3 (33 %)	
Use of cables			
No, n	41 (40 %)	1 (11 %)	p = 0,09 **⁎**
Yes, n	62 (60 %)	8 (89 %)	
Use of Locking Attachment Plate			
No, n	59 (57 %)	5 (56 %)	p = 0,92 **⁎**
Yes, n	44 (43 %)	4 (44 %)	
Use of medial plate			
No, n	101 (98 %)	9 (100 %)	p = 0,67 **⁎**
Yes, n	2 (2 %)	0 (0 %)	
Post-operative weight bearing			
None, n	36 (35 %)	3 (33 %)	p = 0,92 ⁎
Partiel/full, n	67 (65 %)	6 (67 %)	
Post-operative follow-up, days, median (IQR)	136 (299)	147 (152)	p = 0,97 **‡**
Death post-operative, days, median (IQR)	883 (482)	1381 (850)	p = 0,1 **⁑**
Complications			
Infection, n	4 (4 %)	3 (33 %)	**p = 0,001 ⁎**

‡ Wilcoxon Rank Sum test.

† Fishers Exact test.

⁎ Chi^2 test.

⁑ Student's T-test.

## 4 Discussion

With its 165 fractures, this study presents one of the biggest cohorts treated with the VA-LCP Condylar Plate 4.5/5.0 (DePuy Synthes) to our knowledge. We have found it to be a versatile plate applicable to most AO-type fractures (i.e., A-, B-, and C-type fractures) in both the shaft and distal part of the femur, as well as in peri- and interprosthetic fractures. Technically, the implant has been used as both buttress, neutralization, and bridging plates, where the latter dominated numerically. This was properly due to an aging cohort with fragile osteoporotic bone that is poorly managed with lag screws and neutralization plates. Overall, failure was observed in 5,5 % of osteosyntheses, which is comparable to other studies that address failure after osteosynthesis with this implant.^4^^,^^8^, ^9^, ^10^

In the subgroup analysis of risk factors associated with failure, we did not see an association between failure and the length of the plate or the bridging span. This is contrary to the 118 osteosyntheses described by McDonald et al., 2019., where failure was associated with long comminuted fractures and long plates with larger bridging spans.^8^ Inconsistently with Tank et al., 2016, lack of medial support was not found to be a risk factor in this study.^4^ Moreover, the authors warned not to use this plate for AO-type 33C-fractures – a finding we do not retrieve since these fractures were not represented in the failed group.^4^

Consistent with findings by Ricci et al., 2014, this study finds elevated BMI to be significantly elevated in the failure group.^7^ Others have not found this to be a significant risk factor, thus, whether there is a true causal relation is uncertain based on this study retrospective design. However, biomechanically one could speculate that increased weight increases tensile forces on the plate which stresses it towards varus collapse, i.e., the vertical load force, which runs vertically from the hip to the knee joint, is diverted along the anatomic axis of the femur (load-bearing). Metals are ductile, meaning that they stretch out under a given force, and contract back to the starting point when the force is removed. However, studying stress-deformity curves for metals one finds a point where permanent deformation happens. According to others, a long bridge span, long comminuted fractures, and the lack of medial support are risk factors for failure.^4^^,^^8^ Aspects that in our opinion increases the capability of movement across the bridge span, which increases the risk of crossing the point on the stress-deformity curve where permanent deformation happens, which again increases the discrepancy between the vertical and anatomic load axis, i.e., a vicious circle where continuous repetitive load, e.g., during walk, leads to varus collapse until the plate breaks. This understanding seems to be in line with biomechanical studies by Stoffel et al., 2003, who described that longer bridging spans gave the lowest degree of axial and torsional rigidity.^6^ Based on these assumptions, it is our perception that when using the VA-LCP Condylar Plate 4.5/5.0 (DePuy Synthes) one should consider not to make the construct too flexible either by minimizing the bridging span or by inserting a medial buttress plate. However, it is still a fine balance since too rigid constructs seems to be an independent risk factor for non-union.^11^ Thus, the optimal plate length, bridge span, screw density etc. still seem to be unanswered, which leaves us with the basic principles for the use of locking compression plates described by Gautier and Sommer, 2003.^5^

Surprisingly, and contrary to other similar studies, we see a significant overrepresentation of periprosthetic fractures in the failure group. Mittal et al., 2021, described a cohort of 49 interprosthetic fractures, i.e., fracture between a hip and knee arthroplasty, where 3/49 (6 %) failed, which seem comparable to the failure prevalence 8/165 (5 %) in our study.^12^ Furthermore, peri- and interprosthetic fractures account for 50/112 (45 %) in our subgroup analysis, which is considerably more than the numbers in other comparable studies.^4^^,^^9^^,^^10^ Thus, in previous studies the number of these fractures may have been too low to obtain statistically significance (type 2 error).

Post-operative infections were significantly higher in the failure group; however, detailed analysis of patient records show that 2/3 infections were superficial and that the last was an osteosynthesis as a result of an iatrogenic shaft fracture in a 2-two-stage hip arthroplasty revision due to infection. Thus, because of the small numbers in the failure group this finding may be due to chance.

This study distinguish itself by presenting one of the biggest known cohorts treated with VA-LCP Condylar Plate 4.5/5.0 (DePuy Synthes). Furthermore, the unique Danish social security number and access to a nationwide database containing detailed descriptions of all medical records from all hospitals leads to complete follow-up in this study. One of the biggest weaknesses of this study is of course its retrospective design meaning that we cannot address causality. Moreover, based on the relative low frequency of failure there is an increased risk of statistically type 2 error, i.e., the risk of missing an association because the numbers are too small. Finally, incorrect reporting of procedure codes in patient records may have let to loss of patients during inclusion, however, we expect this to be of little importance. The 90-day exclusion criteria was chosen to make this study comparable to the cohort in McDonald et al., 2019.^8^ This led to a relatively large number of excluded patients (n = 27) in the subgroup analysis, however, it is uncertain how these osteosyntheses would have performed if the patients had lived longer, and this may be a source of potential bias in the subgroup analysis.

In conclusion, our study finds the VA-LCP Condylar Plate 4.5/5.0 (DePuy Synthes) to be a versatile plate applicable for alle types of diaphyseal and distal femoral fractures. We did not see an increased risk of failure compared to other previous studies. However, we confirmed that elevated BMI seems to be a risk factor for failure, and found that periprosthetic fractures, too, are overrepresented among implant failures. We acknowledge that the design of this study is retrospective, hence, we cannot draw conclusion on causal matters.

## Financial funding

This research did not receive any specific grant from funding agencies in the public, commercial, or not-for-profit sectors.

## Declaration of interest

None.

## Patient conscent and ethical statement

Due to the retrospective nature of this study, patient conscent was not obtained, however, this study was approved by the Danish National Center for Ethics (Approved: 31-03-2022. File number: 2200031), the Executive Board at Hospital Sønderjylland (Approved: 22-04-2022), registered at The Region of Southern Denmark internal list of scientific studies (Approved: 05-04-2022. File number: 21/43666), and performed in compliance with relevant laws.

## CRediT authorship contribution statement

**Nikolaj Hjort Schmidt:** Methodology, Investigation, Formal analysis, Writing – original draft, Visualization, Project administration. **Lasse Birkelund:** Conceptualization, Methodology, Writing – review & editing. **Jesper Ougaard Schønnemann:** Conceptualization, Methodology, Writing – review & editing, Supervision.

## Figures and Tables

**Table:** 

Patient	Characteristics
#1	Periprosthetic distal femoral fracture (AO 32A2) around Stryker Restoration Modular stem (Vancouver B1). Proximal fixation with only 4 unicortical screws, which later break loose and is documented at follow up 1 month later (failure #1). Treated with amputation of the femur.
#2	Periprosthetic distal femoral fracture (AO 33A2) distal to a cemented hip artroplasty (Vancouver C). Progressive varus mal alignment is documented 2 month post-operatively, and the breach is documented 5 months post-operatively (failure #2). Fixed with a new and longer plate that qualitatively seems to lack medial support, and a new failure is witnessed 2 months post-operatively (failure #3). Next, the fracture is supplied with allograft and fixed with a new plate, and increasing callus formation is seen. At the final inspection in the outpatient clinic, it is uncertain whether the fracture has healed or turned into a hypertrophic non-union.
#3	Distal femoral fracture (AO 33A3) over cemented knee artroplasty (Rorabeck 2). Qualitatively, the osteosynthesis lack medial support. Inspection 6 months post-operatively reveals hypertrophic non-union and bending of the pate (failure #4). The plate is kept in situ and a medial plate is inserted, and the fracture heals with callus formation after this.
#4	Pathological bisphosphonate associated femoral fracture (AO 32A2) around cemented hip artroplasty (Vancouver B1). Qualitatively, the osteosynthesis lack medial support and breaks 3 months post-operatively (failure #5). The plate is removed and a longer stem is inserted.
#5	Distal femoral fracture (AO 33A3) over cemented knee artroplasty (Rorabeck 2). Qualitatively, the fracture is reduced with cables, and a plate 2,5x longer than the fracture length and with a bridging span around 2x the fracture length is inserted. X-ray reveals loosening of the distal screws 1,5-month post-operatively with varus collapse of the fracture (failure #6). The patient is treated conservatively and the fracture heals with varus mal-union.
#6	Distal femoral fracture (AO 33A3) over cemented knee artroplasty (Rorabeck 2). Qualitatively, the osteosynthesis lack medial support; the bridging span is only around 20 % of the plate; and the fracture is 1,5x longer than the bridging span due screw fixation through the fracture. X-ray reveals breaking of the plate 5 month post-operatively (failure #7). The fracture is fixed with a longer plate and heals with callus formation after this.
#7	Diaphyseal femoral fracture (AO 32A2) as a result of iatrogenic injury during revision of infected hip arthroplasty. The plate is fixed to the bone proximally with cables exclusively to allow later insertion of a stem. At the later 2-stage procedure, cables are exchanged with Locking Attachment Plates. Subsequently, an infectious flare-up is suspected, and the patient is suppressed with antibiotics. Later on, the fractures varus collapses and a breach on the plate is seen (failure #8). The failure is fixed with a LCP-DF (DePuy Synthes) and autograft. This plate fails too, and the patient undergoes femoral amputation.
#8	Periprosthetic femoral fracture (AO 32A1) around cemented hip artroplasty (Vancouver B1). Perioperatively, 2 position screws is inserted through the plate and into the fracture just below the stem. The plate breaks in this point 1 month post-operatively (failure #9). The proximal part of the plate is sawn off and a Stryker Restoration Modular stem bypassing the fracture is inserted.

## Appendix 1

Patients assigned with the following surgical procedure codes from 2014 to 2020 were identified in the electronic regional patient record management system.

KNFJ60+KNFJ61+KNFJ62+KNFJ63+KNFJ64+KNFJ65+KNFJ83+KNFJ84+KNFJ85+KNFJ90+KNFJ91+KNFJ92+KNFJ93+KNFJ94+KNFJ95.

## References

[1] Weiss R.J., Montgomery S.M., Al Dabbagh Z., Jansson K.A. (2009). National data of 6409 Swedish inpatients with femoral shaft fractures: stable incidence between 1998 and 2004. Injury.

[2] Hemmann P., Friederich M., Körner D., Klopfer T., Bahrs C. (2021). Changing epidemiology of lower extremity fractures in adults over a 15-year period - a National Hospital Discharge Registry study. BMC Muscoskel Disord.

[3] Jennison T., Divekar M. (2019). Geriatric distal femoral fractures: a retrospective study of 30 day mortality. Injury.

[4] Tank J.C., Schneider P.S., Davis E. (2016). Early mechanical failures of the Synthes variable angle locking distal femur plate. J Orthop Trauma.

[5] Gautier E., Sommer C. (2003). Guidelines for the clinical application of the LCP. Injury.

[6] Stoffel K., Dieter U., Stachowiak G., Gächter A., Kuster M.S. (2003). Biomechanical testing of the LCP--how can stability in locked internal fixators be controlled?. Injury.

[7] Ricci W.M., Streubel P.N., Morshed S., Collinge C.A., Nork S.E., Gardner M.J. (2014). Risk factors for failure of locked plate fixation of distal femur fractures: an analysis of 335 cases. J Orthop Trauma.

[8] McDonald T.C., Lambert J.J., Hulick R.M. (2019). Treatment of distal femur fractures with the DePuy-Synthes variable angle locking compression plate. J Orthop Trauma.

[9] Dang K.H., Armstrong C.A., Karia R.A., Zelle B.A. (2019). Outcomes of distal femur fractures treated with the Synthes 4.5 mm VA-LCP curved Condylar Plate. Int Orthop.

[10] Campana V., Ciolli G., Cazzato G. (2020). Treatment of distal femur fractures with VA-LCP condylar plate: a single trauma centre experience. Injury.

[11] Rodriguez E.K., Zurakowski D., Herder L. (2016). Mechanical construct characteristics predisposing to non-union after locked lateral plating of distal femur fractures. J Orthop Trauma.

[12] Mittal A., Poole W., Crone D. (2021). Interprosthetic femoral fractures managed with modern distal femoral locking plates: 10 years' experience at a UK major trauma centre. Injury.

